# Epstein-Barr Virus Envelope Glycoprotein gp110 Inhibits IKKi-Mediated Activation of NF-κB and Promotes the Degradation of β-Catenin

**DOI:** 10.1128/spectrum.00326-23

**Published:** 2023-04-06

**Authors:** Yingjie Guo, Lingxia Pan, Liding Wang, Shuai Wang, Jiangqin Fu, Wenqi Luo, Kezhen Wang, Xiaoqing Li, Chen Huang, Yintao Liu, Haoran Kang, Qiyuan Zeng, Xiuxia Fu, Zejin Huang, Wanying Li, Yingxin He, Linhai Li, Tao Peng, Haidi Yang, Meili Li, Bin Xiao, Mingsheng Cai

**Affiliations:** a Department of Laboratory Medicine, The Sixth Affiliated Hospital of Guangzhou Medical University, Qingyuan People’s Hospital, State Key Laboratory of Respiratory Disease, The Second Affiliated Hospital, Guangdong Provincial Key Laboratory of Allergy & Clinical Immunology, Guangzhou Medical University, Qingyuan, China; b Department of Clinical Laboratory, Fifth Affiliated Hospital, Southern Medical University, Guangzhou, China; c Institutes of Biology and Medical Sciences, Soochow University, Suzhou, China; d School of Life Sciences, Anhui Medical University, Hefei, China; e State Key Laboratory of Respiratory Disease, Sino-French Hoffmann Institute, School of Basic Medical Science, Guangzhou Medical University, Guangzhou, China; f Guangdong South China Vaccine, Guangzhou, China; g Department of Otolaryngology, Sun Yat-sen Memorial Hospital, Sun Yat-sen University, Guangzhou, China; h Institute of Hearing and Speech-Language Science, Guangzhou Xinhua University, Guangzhou, China; i Department of Pathogenic Biology and Immunology, Sino-French Hoffmann Institute, School of Basic Medical Sciences, Guangzhou Medical University, Guangzhou, China; j Guangzhou Municipal and Guangdong Provincial Key Laboratory of Protein Modification and Degradation, School of Basic Medical Sciences, Guangzhou Medical University, Guangzhou, China; University of Wisconsin-Madison

**Keywords:** EBV gp110, innate immunity, IKKi, β-catenin, IFN-β

## Abstract

Epstein-Barr virus (EBV) infects host cells and establishes a latent infection that requires evasion of host innate immunity. A variety of EBV-encoded proteins that manipulate the innate immune system have been reported, but whether other EBV proteins participate in this process is unclear. EBV-encoded envelope glycoprotein gp110 is a late protein involved in virus entry into target cells and enhancement of infectivity. Here, we reported that gp110 inhibits RIG-I-like receptor pathway-mediated promoter activity of interferon-β (IFN-β) as well as the transcription of downstream antiviral genes to promote viral proliferation. Mechanistically, gp110 interacts with the inhibitor of NF-κB kinase (IKKi) and restrains its K63-linked polyubiquitination, leading to attenuation of IKKi-mediated activation of NF-κB and repression of the phosphorylation and nuclear translocation of p65. Additionally, gp110 interacts with an important regulator of the Wnt signaling pathway, β-catenin, and induces its K48-linked polyubiquitination degradation via the proteasome system, resulting in the suppression of β-catenin-mediated IFN-β production. Taken together, these results suggest that gp110 is a negative regulator of antiviral immunity, revealing a novel mechanism of EBV immune evasion during lytic infection.

**IMPORTANCE** Epstein-Barr virus (EBV) is a ubiquitous pathogen that infects almost all human beings, and the persistence of EBV in the host is largely due to immune escape mediated by its encoded products. Thus, elucidation of EBV’s immune escape mechanisms will provide a new direction for the design of novel antiviral strategies and vaccine development. Here, we report that EBV-encoded gp110 serves as a novel viral immune evasion factor, which inhibits RIG-I-like receptor pathway-mediated interferon-β (IFN-β) production. Furthermore, we found that gp110 targeted two key proteins, inhibitor of NF-κB kinase (IKKi) and β-catenin, which mediate antiviral activity and the production of IFN-β. gp110 inhibited K63-linked polyubiquitination of IKKi and induced β-catenin degradation via the proteasome, resulting in decreased IFN-β production. In summary, our data provide new insights into the EBV-mediated immune evasion surveillance strategy.

## INTRODUCTION

Innate immunity is a relatively conserved nonspecific immunity acquired in the long evolutionary process of organisms and is the first line of host defense against pathogens. Following viral infection of the host, the innate immune system relies on pattern recognition receptors (PRRs) to mediate viral nucleic acid recognition and trigger a series of signaling cascades that induce the expression of type I interferon (IFN-I) and proinflammatory cytokines. Currently, various PRRs have been identified, and cyclic GMP-AMP synthase (cGAS) and RIG-I-like receptors (RLRs) are the typical representatives of DNA sensors and RNA sensors, respectively (1, 2).

After binding to viral nucleic acid, these PRRs recruit downstream adaptor proteins to further initiate many kinases and activate downstream transcription factors. Specifically, RIG-I recognizes cytoplasmic double-stranded RNA (dsRNA) or single-stranded RNA (ssRNA) containing 5′-triphosphate (5′-ppp). For the replication product of DNA virus in the cytoplasm, poly(dA-dT) DNA is transcribed by RNA polymerase III (Pol-III) to form 5′-ppp-dsRNA, which can also activate the RLR pathway and trigger the host’s IFN-I response (3, 4). RIG-I recognizes viral RNA and changes conformation to interact with mitochondrial antiviral signaling protein (MAVS) via the caspase recruitment domain (CARD)-CARD interaction, and the latter recruits tumor necrosis factor (TNF) receptor-associated factor 3 (TRAF3), which further recruits the activated kinases TANK binding kinase 1 (TBK1) and inhibitor of NF-κB kinase (IKKi) to phosphorylate IFN regulatory factor 3 (IRF3) and/or IRF7. IRFs then transfer to the nucleus to induce the antiviral response (5). Additionally, IKKi is critical for NF-κB activation, which mediates p65 (Ser536) phosphorylation in response to proinflammatory signals and viral infection (6, 7). Concerning viral DNA sensing, cGAS catalyzes the synthesis of cGAMP, which binds to stimulator of IFN genes (STING) to induce its activation, then STING recruits TBK1 and IRF3, causing TBK1 to phosphorylate IRF3 and stimulate downstream IFN-I production (8, 9). IRF3 also associates with the transcriptional coactivator CREB-binding protein (CBP) and/or p300. This complex then binds to the positive regulatory element PRDIII-I of the IFN-β promoter to enhance its production (10–12). Moreover, β-catenin, an important regulator of the Wnt signaling pathway, can also promote CBP and p300 to recruit IRF3 to the IFN-β promoter by forming the β-catenin-IRF3-CBP/p300 complex (13, 14).

Epstein-Barr virus (EBV), the ubiquitous human gammaherpesvirus, was discovered in 1964 from tissue cell culture of Burkitt lymphoma in African children (15). EBV is mainly transmitted through saliva, but it also can be transmitted through sexual contact, and in a few cases, EBV can be transmitted to newborns through lactation (16). EBV predominantly infects epithelial cells, B cells, and macrophages, and dendritic cells also play an important role in infection (17). After the initial infection, EBV sustains a latent state in infected host cells for a long time and exists in the form of episomes, which are highly associated with proliferative diseases, such as Burkitt’s lymphoma, Hodgkin’s lymphoma, and non-Hodgkin’s lymphoma as well as epithelial cancers, including nasopharyngeal carcinoma (18). Under specific conditions, the viral BZLF1 protein (also known as Zta) is expressed and induces EBV reactivation, leading to the production of progeny virions and spread of EBV (19).

To complete the life cycle, EBV gradually forms a unique survival strategy against the host’s immune response in the process of long-term resistance to innate immunity. EBV is known to encode more than 80 viral proteins, some of which have been shown to be involved in the course of antagonizing host immunity. For example, our previous study showed that the tegument protein BFRF1 suppresses IFN-β activity by binding to IKKi to inhibit the activation of IRF3 (20). The protein kinase BGLF4 interacts with and phosphorylates IRF3 to weaken its ability to associate with the IFN-β promoter (21). LMP1 interplays with tyrosine kinase 2 to restrain the phosphorylation of signal transducer and activator of transcription 1 (STAT1) and STAT2 to block the IFN-mediated antiviral response (22). LMP1 also induces the expression of some antiapoptotic proteins, such as survivin, A20, and BCL-2, to facilitate viral infection (23). In addition, we also disclosed that the membrane protein BGLF2 interacts with the NF-κB subunits p65 and p50 to hamper the phosphorylation (Ser536) and nuclear translocation of p65 (24).

The EBV *BALF4*-encoded envelope glycoprotein gp110, a late viral gene product expressed during the lytic phase of EBV infection, is primarily located in the cytoplasm, endoplasmic reticulum, internal or external nuclear membrane, plasma membrane, and cell surface (25–27). gp110 participates in EBV entry into host target cells through endocytosis and/or direct cell fusion (28), which is also critical for viral assembly and maturation (29–31). Additionally, gp110 can serve as a target for antibody-dependent cell-mediated cytotoxicity (32), and it is a critical virulence factor for EBV infection of non-B lymphocytes, which determines EBV infectivity and its virus tendency (30, 33). However, it is unknown whether gp110 is involved in the regulation of the host IFN-I response.

In the present study, we showed that gp110 is a negative regulator of host IFN-I production. Mechanistically, gp110 interacts with IKKi to attenuate IKKi-mediated NF-κB activation. Furthermore, gp110 binds to β-catenin, an important regulator of the Wnt signaling pathway, to promote its proteasomal degradation through K48-linked polyubiquitination and reduce the formation of the β-catenin-IRF3-CBP/p300 conformer, resulting in decreased IFN-β production. These findings reveal a novel mechanism of EBV immune evasion during the course of lytic infection.

## RESULTS

### EBV gp110 downregulates Sendai virus (SeV)-mediated antiviral signaling.

**FIG 1:**
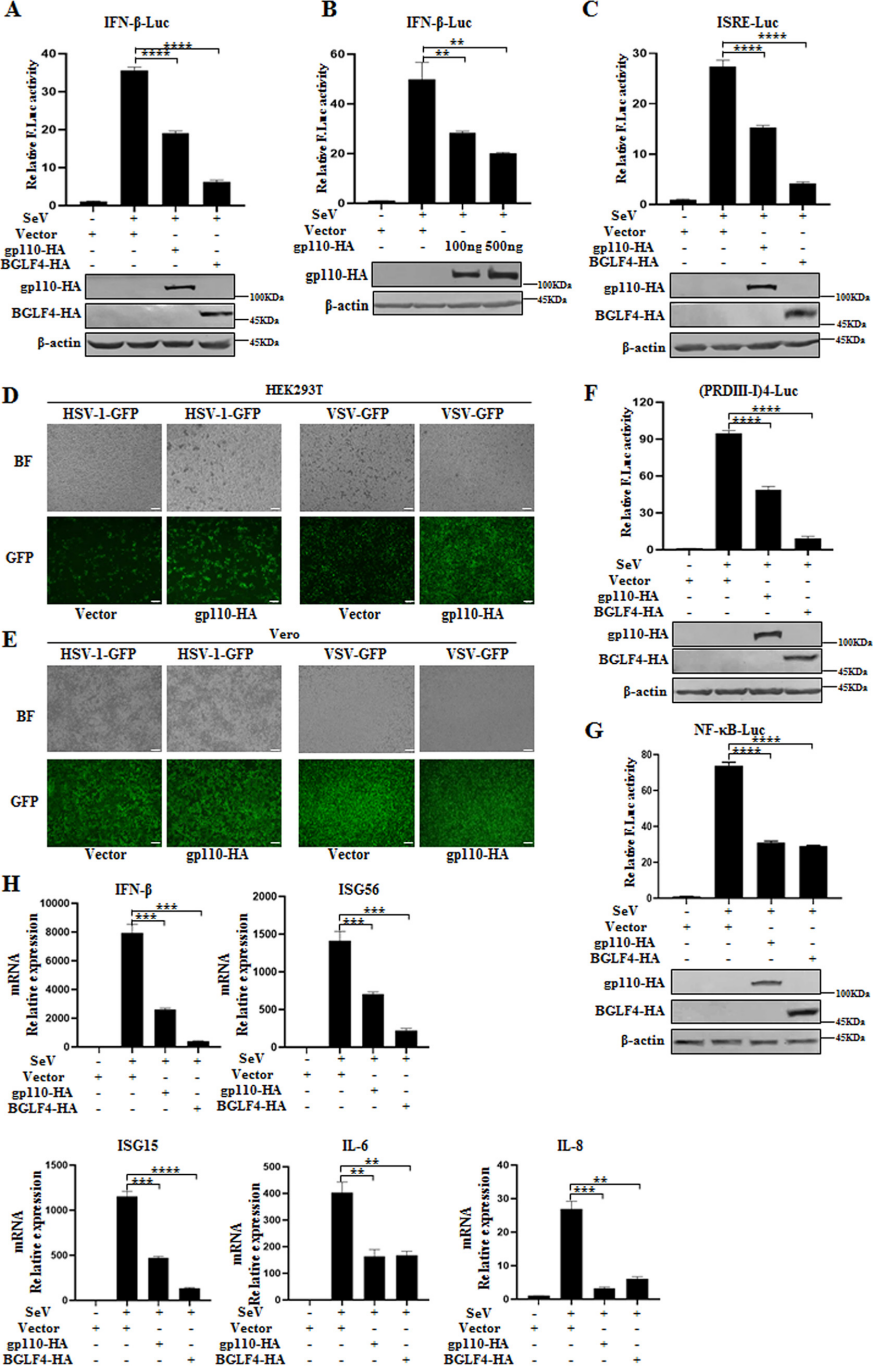
EBV gp110 suppresses host antiviral immunity. (A, C, F, and G) One hundred nanograms of IFN-β-Luc (A), ISRE-Luc (C), (PRDIII-I)4-Luc (F), or NF-κB-Luc (G) was cotransfected with 10 ng of the internal reference pRL-TK into HEK293T cells along with the expression plasmid (500 ng) of gp110-HA, BGLF4-HA, or HA vector. At 24 h posttransfection, cells were infected with or without 100 HAU/mL SeV for 16 h, cell lysates were collected, and luciferase activity was detected by DLR. (B) HEK293T cells were cotransfected with IFN-β-Luc and RL-TK along with the indicated amounts (100 and 500 ng) of gp110-HA expression plasmid or HA vector. DLR was performed as indicated in A. The transfected proteins were examined by WB with mouse anti-HA MAb, and β-actin was used as the loading control. (D and E) HEK293T cells (D) or Vero cells (E) were transfected with gp110-HA expression plasmid or HA vector, and at 24 h posttransfection, cells were infected with HSV-1-GFP or VSV-GFP at a multiplicity of infection (MOI) of 1 for 24 h. Viral replication was observed and photographed using a fluorescence microscope. The GFP fluorescence reflects the replication of the virus, and BF represents brightfield. All scales indicate 200 μM. (H) HeLa cells were transfected with the expression plasmid of gp110-HA, BGLF4-HA, or HA vector, and at 24 h posttransfection, cells were infected with or without 100 HAU/mL SeV for 16 h. Cells were then collected, total RNA was extracted, and real-time qPCR was used to detect the mRNA expression of *IFN-β*, *ISG15*, *ISG56*, *IL-6*, *IL-8*, and *GAPDH*. Data are presented as mean ± SD, and statistical analysis was performed using a Student’s *t* test; **, 0.001 < *P* < 0.01; ***, 0.0001 < *P* < 0.001; ****, *P* < 0.0001.

To identify the possibility of EBV gp110 in regulating the host antiviral response, the IFN-β luciferase reporter plasmid and gp110 expression plasmid or positive-control BGLF4 expression plasmid, which is reported to inhibit IFN-β production (21), were cotransfected into human embryonic kidney 293T (HEK293T) cells. Cells were then infected with the effective inducer of the RLR pathway SeV (34). The effects of gp110 on IFN-β promoter activity were detected by dual-luciferase reporter (DLR) assays. Compared with the positive control BGLF4, the expression of gp110 significantly inhibited the activity of the IFN-β promoter (Fig. 1A); its inhibitory effect was also dose dependent (Fig. 1B).

After virus invasion, host innate immunity is activated through a series of cascade reactions, which eventually stimulate the transcription factor complex to bind to IFN-stimulated response elements (ISREs), leading to the activation of hundreds of IFN-stimulated genes (ISGs) and inflammatory cytokines to execute their antiviral roles (35). Next, DLR assays were performed using the ISRE luciferase reporter, and results showed that the activity of the ISRE promoter was also inhibited in the presence of gp110 (Fig. 1C).

To continue to analyze whether gp110 can promote viral propagation, vector or gp110 expression plasmid was transfected into IFN-I-producing HEK293T cells or IFN-I-deficient Vero cells, which are also used by other groups to prove related findings (36, 37). Cells were then infected with herpes simplex virus 1-green fluorescent protein (HSV-1-GFP; DNA virus) or vesicular stomatitis virus-GFP (VSV-GFP; RNA virus), and GFP fluorescence expression was detected to monitor virus infection. As shown in Fig. 1D, overexpression of gp110 in HEK293T cells obviously increased the cytopathic effect and viral fluorescence when cells were infected with HSV-1-GFP or VSV-GFP, but this difference was almost nonexistent in Vero cells, as the latter is deficient in the IFN-I response (Fig. 1E) (36), indicating that gp110 has the ability to weaken the host antiviral response, thereby promoting virus replication.

The expression of IFN-I is mediated by the activation of distinct potential transcriptional regulatory factors, including NF-κB, IRF3, IRF7, and other coactivators (38). To investigate which branch of IFN-β production is impeded by gp110, DLR assays were performed. SeV infection significantly activated the promoter activities of (PRDIII-I)4-luciferase ([PRDIII-I]4-Luc) and NF-κB-Luc, but their activities were suppressed in the presence of gp110 (Fig. 1F and G). Collectively, these results indicate that gp110 inhibits SeV-mediated IFN-β production through both the IRF3 branch and the NF-κB branch.

To determine whether gp110 affects the mRNA level of IFN-β and its downstream ISGs or NF-κB-regulated inflammatory cytokines, vector, gp110 expression plasmid, or BGLF4 expression plasmid was transfected into HEK293T cells and infected with SeV, then real-time quantitative PCR (qPCR) was conducted. As shown in Fig. 1H, mRNA expression of *IFN-β*, *ISG15*, *ISG56*, *IL-6*, and *IL-8* was hampered when gp110 was expressed. These results suggest that gp110 can suppress RLR pathway-mediated antiviral immunity.

### Knockdown of gp110 during EBV infection enhances host antiviral immunity.

**FIG 2:**
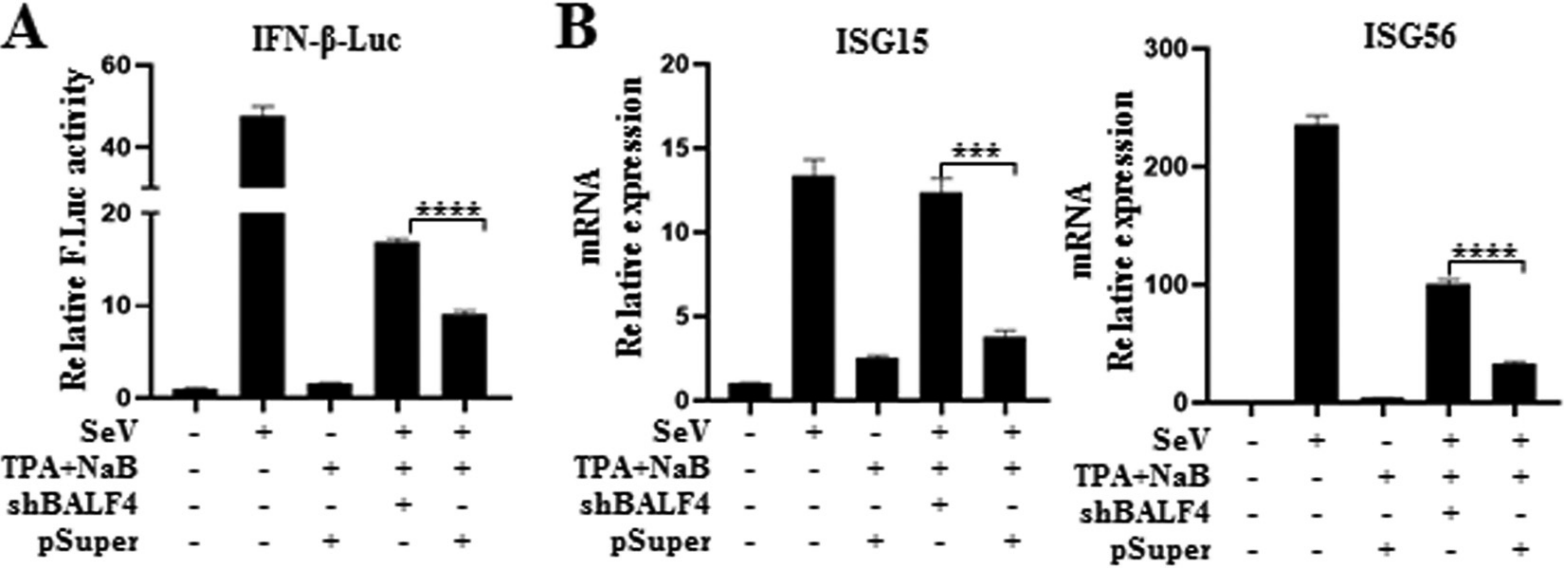
Effect of gp110 knockdown on SeV-triggered antiviral immunity. (A) One hundred nanograms of IFN-β-Luc was cotransfected with 10 ng of the internal reference pRL-TK into Hone1-EBV cells together with the expression plasmid (500 ng) of shBALF4 or pSuper vector, and at 24 h posttransfection, TPA (40 ng/mL) and NaB (3 mM) were added to the cells for 24 h to induce EBV lytic infection. Cells were then infected with or without 100 HAU/mL SeV for 16 h, cell lysates were collected, and luciferase activity was detected by DLR. (B) Hone1-EBV cells were transfected with the expression plasmid (500 ng) of shBALF4 or pSuper vector, and at 24 h posttransfection, TPA (40 ng/mL) and NaB (3 mM) were added to the cells for 24 h to induce EBV lytic infection. Cells were then infected with or without 100 HAU/mL SeV for 16 h and collected, and total RNA was extracted. Real-time qPCR was used to detect the mRNA expression of *ISG15*, *ISG56*, and *GAPDH*. Data are presented as mean ± SD, and statistical analysis was performed using a Student’s *t* test; ***, 0.0001 < *P* < 0.001; ****, *P* < 0.0001.

To examine the role of gp110 in the innate antiviral response during EBV infection, an RNA interference (RNAi) knockdown strategy was used, as applied in our previous studies (20, 24). First, a gp110-knockdown expression plasmid shBALF4 was constructed (39), which can effectively inhibit the expression of gp110 in EBV lytic-infected Hone1 cells treated with 12-*O*-tetradecanoylphorbol-13-acetate (TPA) and sodium butyrate (NaB). Then, lytic EBV infection in Hone1-EBV cells transfected with the IFN-β luciferase reporter and the gp110-knockdown expression plasmid shBALF4 or pSuper vector was induced, and cells were stimulated with SeV to activate antiviral immunity. DLR assays were then performed. Compared with the pSuper vector, the promoter activity of IFN-β-Luc increased when gp110 was knocked down in EBV^+^ Hone1 cells (Fig. 2A). To further validate the above results, the expression of antiviral genes at the mRNA level after knockdown of gp110 was detected. As expected, the expression of *ISG15* and *ISG56* was increased when gp110 was knocked down (Fig. 2B). These results demonstrate that gp110 can downregulate RLR pathway-mediated IFN-β production and negatively regulate antiviral immunity during EBV lytic replication.

### gp110 interacts with IKKi.

**FIG 3:**
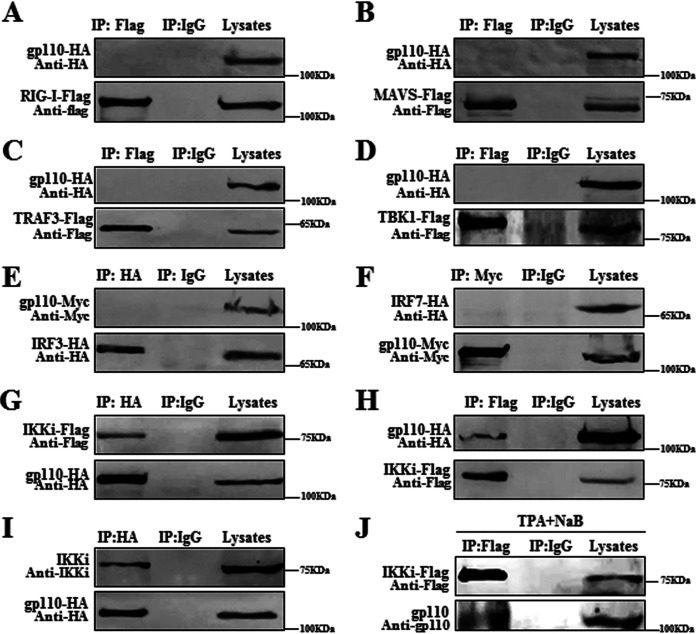
gp110 is associated with IKKi. (A to I) HEK293T cells were individually transfected with gp110-HA expression plasmid (I) or cotransfected with the expression plasmid combination of gp110-HA/RIG-I-Flag (A), gp110-HA/MAVS-Flag (B), gp110-HA/TRAF3-Flag (C), gp110-HA/TBK1-Flag (D), gp110-Myc/IRF3-HA (E), gp110-Myc/IRF7-HA (F), or gp110-HA/IKKi-Flag (G and H). At 24 h posttransfection, cells were collected and lysed. The samples were then used for Co-IP analysis with mouse anti-Flag MAb (A to D and H), mouse anti-HA MAb (E, G, and I), mouse anti-Myc MAb (F), or nonspecific IgG. (J) Hone1-EBV cells were transfected with IKKi-Flag expression plasmid. At 24 h posttransfection, cells were treated with TPA (40 ng/mL) and NaB (3 mM) for 24 h to induce EBV lytic infection, and cells were collected and lysed. The sample was then used for Co-IP analysis with mouse anti-Flag MAb or nonspecific IgG. Immunoprecipitated proteins were then resolved by 10% SDS-PAGE, and WB was performed with anti-Flag, anti-HA, or anti-Myc (A to J). Rabbit anti-IKKi pAb and mouse anti-gp110 MAb were used to detect the expression of endogenous IKKi (I) and gp110 (J), respectively.

Viral proteins bind to some signaling molecules to prevent their functions and evade IFN-mediated antiviral responses (40). To further investigate the molecular mechanism of gp110 inhibiting the IFN-β signal pathway, gp110 expression plasmid was cotransfected with RIG-I, MAVS, TRAF3, IKKi, TBK1, IRF3, or IRF7 expression plasmid, and cells were collected and lysed for coimmunoprecipitation (Co-IP) assays. As shown in Fig. 3, overexpressed gp110 did not coprecipitate with RIG-I, MAVS, TRAF3, TBK1, IRF3, or IRF7 (Fig. 3A to F), but it was associated with IKKi (Fig. 3G and H), and overexpression of gp110 could interact with endogenous IKKi (Fig. 3I). To further verify whether gp110 interacts with endogenous IKKi under physiological conditions, IKKi expression plasmid-transfected Hone1-EBV cells were treated with TPA and NaB to induce EBV lytic infection, and Co-IP showed that gp110 could be immunoprecipitated by IKKi, suggesting that gp110 can interact with IKKi during EBV lytic infection (Fig. 3J).

### gp110 inhibits K63-linked polyubiquitination of IKKi and impairs its kinase function.

**FIG 4:**
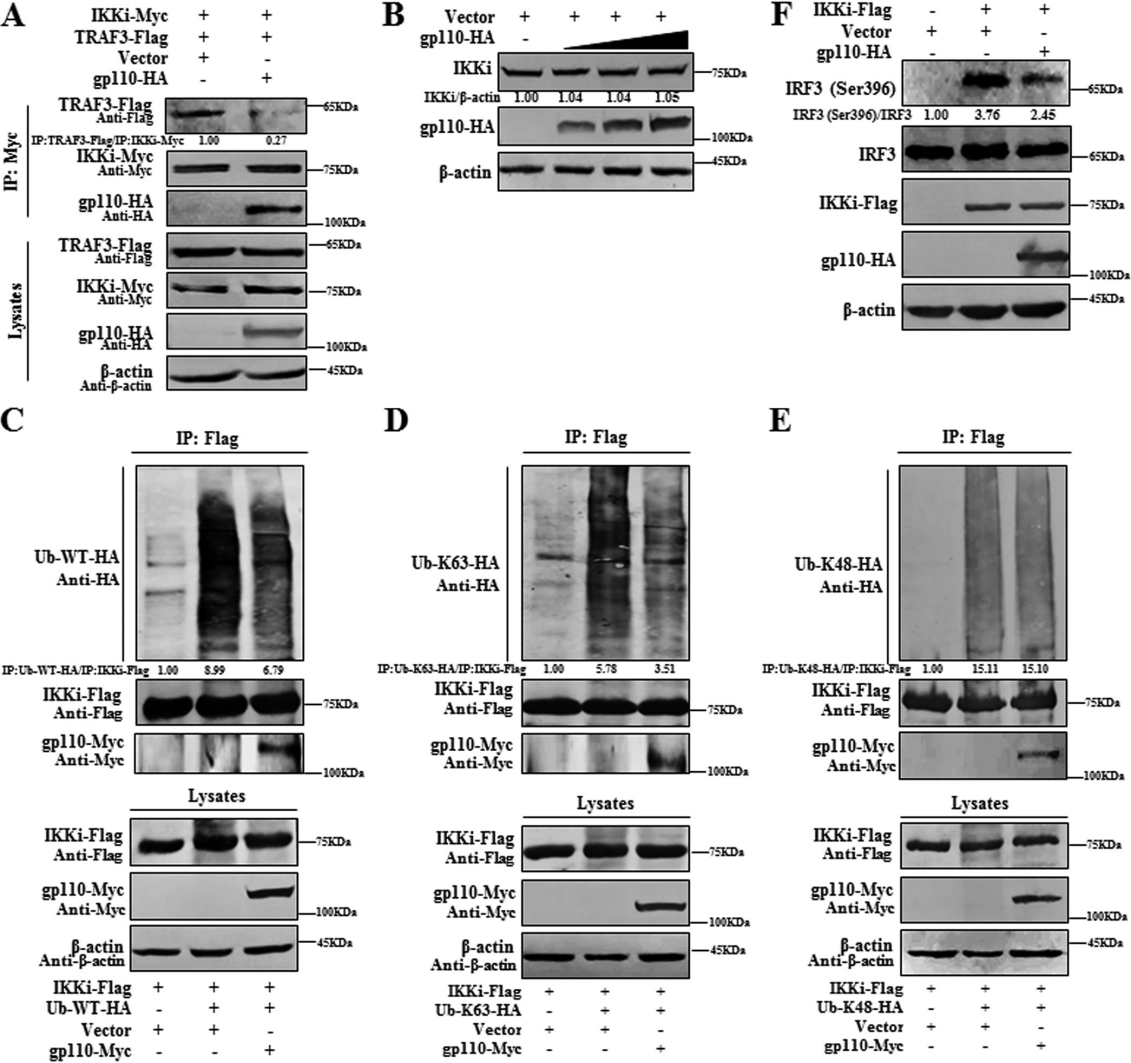
gp110 inhibits K63-linked polyubiquitination of IKKi and impairs IKKi-activated IRF3 phosphorylation. (A) gp110-HA expression plasmid or vector was cotransfected with the plasmid combination of TRAF3-Flag/IKKi-Myc into HEK293T cells. At 24 h posttransfection, cells were collected and lysed. The samples were then used for Co-IP analysis with mouse anti-Myc MAb. (B) HEK293T cells were transfected with gp110-HA expression plasmid or vector. At 24 h posttransfection, the cell lysates were collected for WB. Here, to avoid the influence on the results caused by the difference in the amount of plasmid transfection in each experimental well, empty plasmid was added to ensure the total amount of plasmids in the transfection system was consistent in each group when the relevant dose gradient experiments were performed, and a similar approach was used in the following related experiments. (C to E) HEK293T cells were cotransfected with the expression plasmids of IKKi-Flag and gp110-Myc or vector along with WT ubiquitin (Ub-WT-HA; C), K63 (Ub-K63-HA; D), or K48 (Ub-K48-HA; E) expression plasmid. At 24 h posttransfection, cells were collected and lysed. The samples were then used for Co-IP analysis with mouse anti-Flag MAb. (F) IKKi-Flag expression plasmid was cotransfected with gp110-HA or vector into HEK293T cells. At 24 h posttransfection, the cell lysates were collected for WB. All WB was performed using the indicated Abs, and the gray analysis was calculated using ImageJ.

TRAF3 is a key antiviral signaling molecule that functions as a ubiquitin (Ub) ligase upstream of TBK1-IKKi (41–43). To probe whether the gp110 and IKKi interaction can disrupt the complex formation of TRAF3 and IKKi and inhibit TRAF3-dependent polyubiquitination of IKKi, HEK293T cells were cotransfected with IKKi and TRAF3 expression plasmids along with vector or gp110 expression plasmid, and Co-IP was performed. Compared with the vector, overexpression of gp110 disrupted the interaction between TRAF3 and IKKi (Fig. 4A), suggesting that gp110 can block the formation of the TRAF3-IKKi complex. To analyze whether the gp110 and IKKi interaction can impact the expression of IKKi, vector or different doses of gp110 expression plasmid were transfected into HEK293T cells, and Western blotting (WB) showed that gp110 did not affect the expression of IKKi (Fig. 4B).

Ubiquitination is a common posttranslational modification (44). Several regulatory molecules in the IFN-β signaling pathway require ubiquitination, especially the ubiquitination and deubiquitination of K48 and K63, which play a key role in the activation of IFN-β production (45, 46). Studies have shown that K63-linked polyubiquitination of TBK1-IKKi promotes the activation of IRF3 (42, 47). To explore whether the gp110 and IKKi interaction influences the polyubiquitination of IKKi, HEK293T cells were cotransfected with IKKi expression plasmid and vector or gp110 expression plasmid along with wild-type (WT) ubiquitin or its K48 or K63 ubiquitin expression plasmid, then Co-IP was performed. We observed that gp110 impeded the polyubiquitination of IKKi with the expression of WT ubiquitin (Fig. 4C) and K63 ubiquitin (Fig. 4D), but not K48 ubiquitin (Fig. 4E). Therefore, the binding of gp110 to IKKi may mask the ubiquitination region of IKKi, resulting in attenuated TRAF3-dependent polyubiquitination regulation of IKKi.

K63-linked polyubiquitination of IKKi can disturb the activation of its downstream IRF3 substrate (48), and studies have shown that virus-encoded proteins hamper IFN-β production by inhibiting IKKi kinase function (20, 49). To inspect the effect of gp110 on IKKi kinase activity, gp110 expression plasmid or vector was transfected into HEK293T cells along with IKKi expression plasmid to activate endogenous IRF3. We observed that gp110 could inhibit IKKi-activated IRF3 (Ser396) phosphorylation (Fig. 4F). Accordingly, gp110 may inhibit IFN-I production by constraining K63-linked polyubiquitination and its kinase activity of IKKi.

### gp110 does not affect the phosphorylation, dimerization, or nuclear translocation of IRF3.

**FIG 5:**
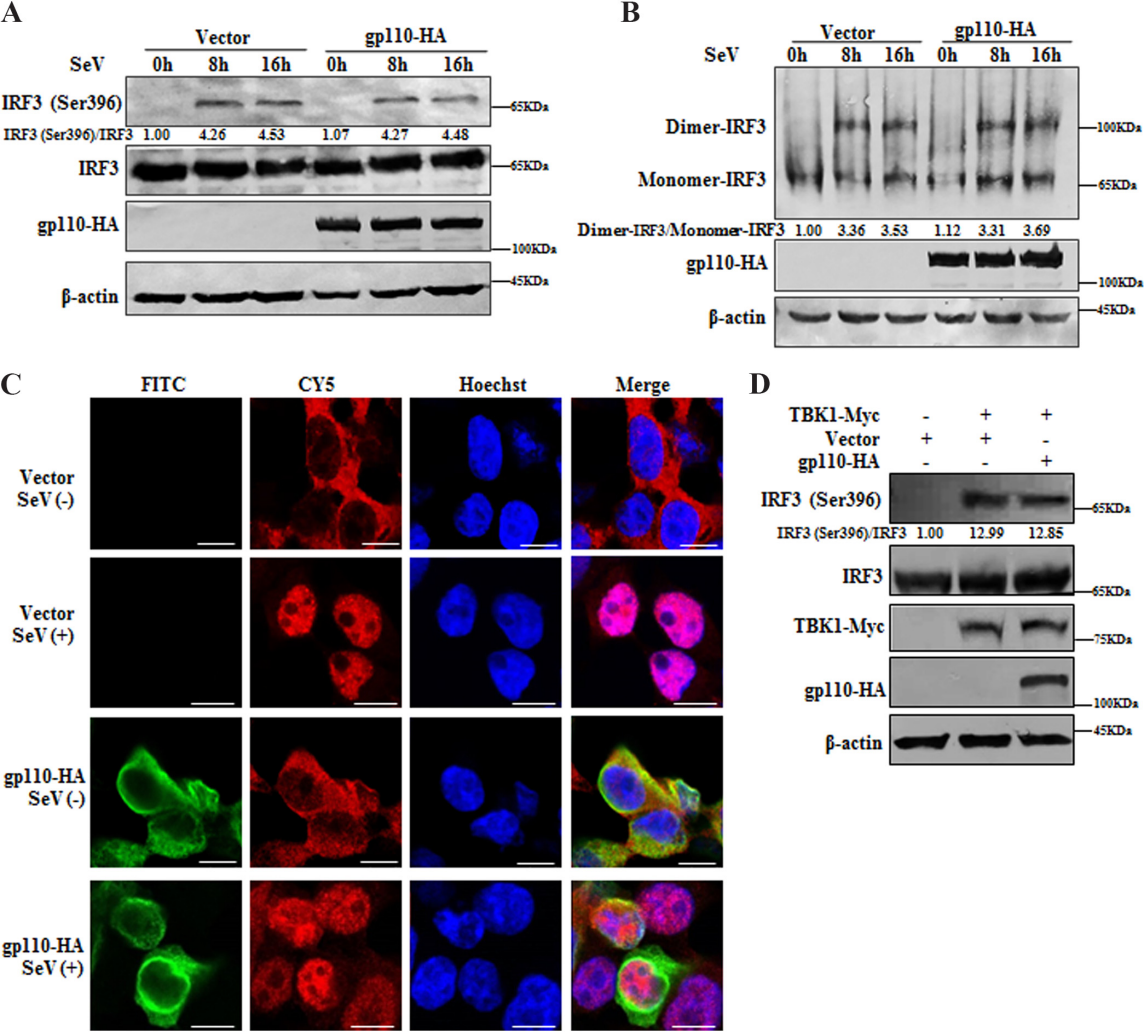
gp110 does not affect SeV-mediated IRF3 phosphorylation, dimerization, or nuclear translocation. (A) HEK293T cells were transfected with gp110-HA expression plasmid or vector. At 24 h posttransfection, cells were infected with or without 100 HAU/mL SeV for 0, 8, or 16 h, and cell lysates were collected for WB with rabbit anti-IRF3 pAb and mouse anti-HA MAb. Rabbit anti-IRF3 (Ser396) pAb was used to detect the phosphorylation of IRF3, and β-actin was used as the loading control. (B) HEK293T cells were transfected with gp110-HA expression plasmid or vector. At 24 h posttransfection, cells were infected with or without 100 HAU/mL SeV for 0, 8, or 16 h, cell lysates were collected and subjected to native PAGE, and WB was performed with mouse anti-HA MAb. The dimer and monomer of IRF3 were detected by rabbit anti-IRF3 pAb, and β-actin was used as the loading control. (C) HeLa cells were transfected with gp110-HA expression plasmid or vector. At 24 h posttransfection, cells were infected with or without 100 HAU/mL SeV for 16 h. Cells were then fixed, and IFA was performed with the primary Abs mouse anti-HA MAb and rabbit anti-IRF3 pAb and secondary Abs FITC-conjugated goat anti-mouse IgG (green) and Cy5-conjugated goat anti-rabbit IgG (red). The nuclei were stained with Hoechst 33342 (blue), and images were acquired with a laser-scanning confocal microscope. All scales indicate 10 μM. (D) HEK293T cells were transfected with gp110-HA expression plasmid or vector along with TBK1-Myc expression plasmid to activate the phosphorylation of IRF3. At 24 h posttransfection, cell lysates were collected for WB analysis with rabbit anti-IRF3 pAb, mouse anti-Myc MAb, and mouse anti-HA MAb. Rabbit anti-IRF3 (Ser396) was used to detect the phosphorylation of IRF3, and β-actin was used as the loading control. The gray analysis was calculated using ImageJ.

Following activation of the IFN-β signaling pathway, the TBK1 and IKKi kinases phosphorylate IRF3, followed by its dimerization and translocation into the nucleus, which is critical for IFN-β transcription. Ser396 is a phosphorylated target after virus infection and plays an important role in IRF3 activation (50). To test whether gp110 can interfere with the phosphorylation and formation of the IRF3 dimer, HEK293T cells were transfected with vector or gp110 expression plasmid and infected with SeV for the indicated amounts of time. Unexpectedly, SeV infection induced the phosphorylation (Ser396) and dimerization of IRF3, but this was not restrained in the presence of gp110 (Fig. 5A and B).

To determine whether gp110 can block the nuclear accumulation of IRF3, HeLa cells, which are suitable and widely used to observe the nuclear translocation of target proteins as its nucleus is relatively large (20, 24, 51–66), were transfected with vector or gp110 expression plasmid and infected with SeV. Immunofluorescence assays (IFAs) showed that in mock-treated cells, IRF3 was localized in the cytoplasm, and IRF3 was transferred to the nucleus when cells were infected with SeV. However, ectopic expression of gp110 had no impact on the nuclear trafficking of IRF3 (Fig. 5C). TBK1 and IKKi are reported to act redundantly in activating IFN-I production (67, 68), and gp110 had no effect on the function of TBK1 mediating IRF3 phosphorylation (Fig. 5D), indicating that gp110 may inhibit the phosphorylation of IRF3 by targeting IKKi in a uniquely dependent manner.

### gp110 facilitates the degradation of β-catenin.

**FIG 6:**
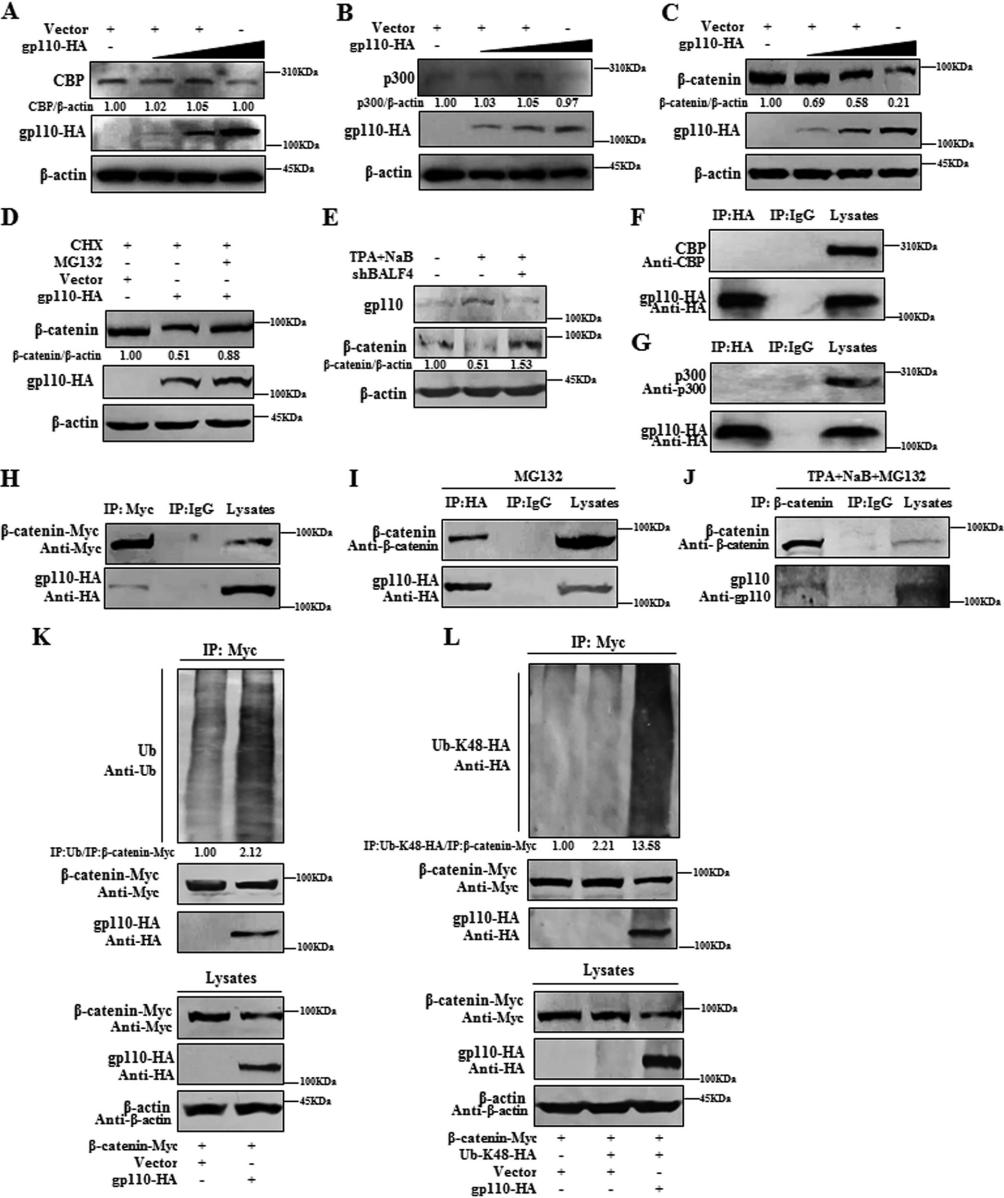
gp110 degrades β-catenin through the proteasome system. (A to C) HEK293T cells were transfected with the indicated concentrations (0, 500, 1,000, and 1,500 ng) of gp110-HA expression plasmid or vector. At 24 h posttransfection, cell lysates were collected, and the expression of endogenous CBP (A), p300 (B), and β-catenin (C) was detected by WB with mouse anti-CBP pAb, mouse anti-p300 pAb, rabbit anti-β-catenin pAb, and mouse anti-HA MAb, and β-actin was used as the loading control. (D) HEK293T cells were transfected with gp110-HA expression plasmid or vector. At 24 h posttransfection, cells were treated with CHX (50 μg/mL) alone or together with or without MG132 (20 μM) for 4 h. Cells were collected for WB, as indicated in C. (E) Hone1-EBV cells were transfected with the expression plasmid of shBALF4. At 24 h posttransfection, cells were treated with or without TPA (40 ng/mL) and NaB (3 mM) for 24 h to induce EBV lytic infection. Cell lysates were collected for WB with mouse anti-gp110 MAb and rabbit anti-β-catenin pAb, and β-actin was used as the loading control. (F to I) HEK293T cells were individually transfected with gp110-HA expression plasmid (F, G, and I) or cotransfected with the expression plasmid combination of gp110-HA/β-catenin-Myc (H). At 24 h posttransfection, cells were treated with or without MG132 (20 μM) for 4 h to inhibit gp110-mediated β-catenin degradation, and cells were collected and lysed. The samples were then used for Co-IP analysis with mouse anti-HA MAb (F, G, and I), mouse anti-Myc MAb (H), or nonspecific IgG. (J) Hone1-EBV cells were stimulated with TPA (40 ng/mL) and NaB (3 mM) for 24 h to induce lytic EBV infection, then MG132 (20 μM) was added for 4 h, and the cells were collected and lysed. The samples were then used for Co-IP analysis with rabbit anti-β-catenin pAb or nonspecific IgG. (K and L) HEK293T cells were cotransfected with the expression plasmids of β-catenin-Myc and gp110-HA or vector along with or without K48 ubiquitin expression plasmid Ub-K48-HA. At 24 h posttransfection, cells were collected and lysed. The samples were then used for Co-IP analysis with mouse anti-Myc MAb. Immunoprecipitated proteins were then resolved by 10% SDS-PAGE, and WB was performed with mouse anti-HA MAb and mouse anti-Myc MAb. Mouse anti-CBP pAb, mouse anti-p300 pAb, rabbit anti-β-catenin pAb, mouse anti-gp110 MAb, and rabbit anti-Ub pAb were used to detect the expression of endogenous CBP (F), p300 (G), β-catenin (I and J), gp110 (J), and ubiquitin (K), respectively. The gray analysis was calculated using ImageJ.

After translocation of IRF3 into the nucleus, CBP and/or p300 are recruited, and this complex then binds to PRDIII-I on the IFN-β promoter to enhance IFN-β production (10–12). In this process, β-catenin functions through the formation of a complex between IRF3 and CBP/p300 to regulate IFN-I production (69–71), which promotes the recruitment of p300 to the IFN-β promoter through IRF3 (72). To analyze whether gp110 can impact the expression of CBP, p300, or β-catenin, vector or gp110 expression plasmid was transfected into HEK293T cells, and WB showed that gp110 had no effect on the expression of CBP (Fig. 6A) or p300 (Fig. 6B), but it could degrade endogenous β-catenin in a dose-dependent manner (Fig. 6C). Addition of the proteasome inhibitor MG132, a peptide aldehyde that blocks the proteolytic activity of the proteasome, could restore this degradation (Fig. 6D), suggesting that gp110 promotes the proteasomal degradation of β-catenin. To further verify this result under physiological conditions, pSuper vector or shBALF4 expression plasmid was transfected into Hone1-EBV cells, and cells were then induced into lytic infection with TPA and NaB. Compared with the control, the expression of β-catenin was increased when gp110 was knocked down (Fig. 6E), indicating that gp110 indeed can induce proteasome-mediated degradation of β-catenin during EBV infection.

To deeply investigate the gp110-mediated proteasomal degradation of β-catenin, gp110 expression plasmid was individually transfected or cotransfected with β-catenin expression plasmid into HEK293T cells, and Co-IP showed that the overexpressed gp110 could not interact with endogenous CBP (Fig. 6F) or p300 (Fig. 6G), but it was associated with overexpressed β-catenin (Fig. 6H). To explore the relationship between gp110 and endogenous β-catenin, HEK293T cells were transfected with gp110 expression plasmid and treated with the proteasome inhibitor MG132 to inhibit gp110-mediated degradation of β-catenin, as the expression of endogenous β-catenin must be much lower than that of plasmid transfection. The results showed that overexpression of gp110 could interact with endogenous β-catenin (Fig. 6I). In addition, Hone1-EBV cells were treated with TPA and NaB to induce lytic infection and incubated with MG132. Co-IP experiments confirmed that gp110 could interact with endogenous β-catenin during EBV lytic infection (Fig. 6J).

Generally, proteasome-mediated degradation of specific proteins is first initiated by labeling with ubiquitin, and polyubiquitination at K48 plays a main role in the degradation and regulation of protein stability (73). To continue to dissect the mechanism of the gp110 and β-catenin interaction and its induced degradation via the proteasome, HEK293T cells were cotransfected with the expression plasmids of β-catenin and gp110 or vector, and Co-IP displayed that gp110 could increase the polyubiquitination of β-catenin (Fig. 6K). Moreover, gp110 promoted K48-linked polyubiquitination of β-catenin when the K48 ubiquitin expression plasmid was cotransfected with the plasmids mentioned above (Fig. 6L), which further confirms that gp110 mediates the degradation of β-catenin through the proteasomal pathway.

### β-Catenin-mediated IFN-β production is antagonized by gp110.

**FIG 7:**
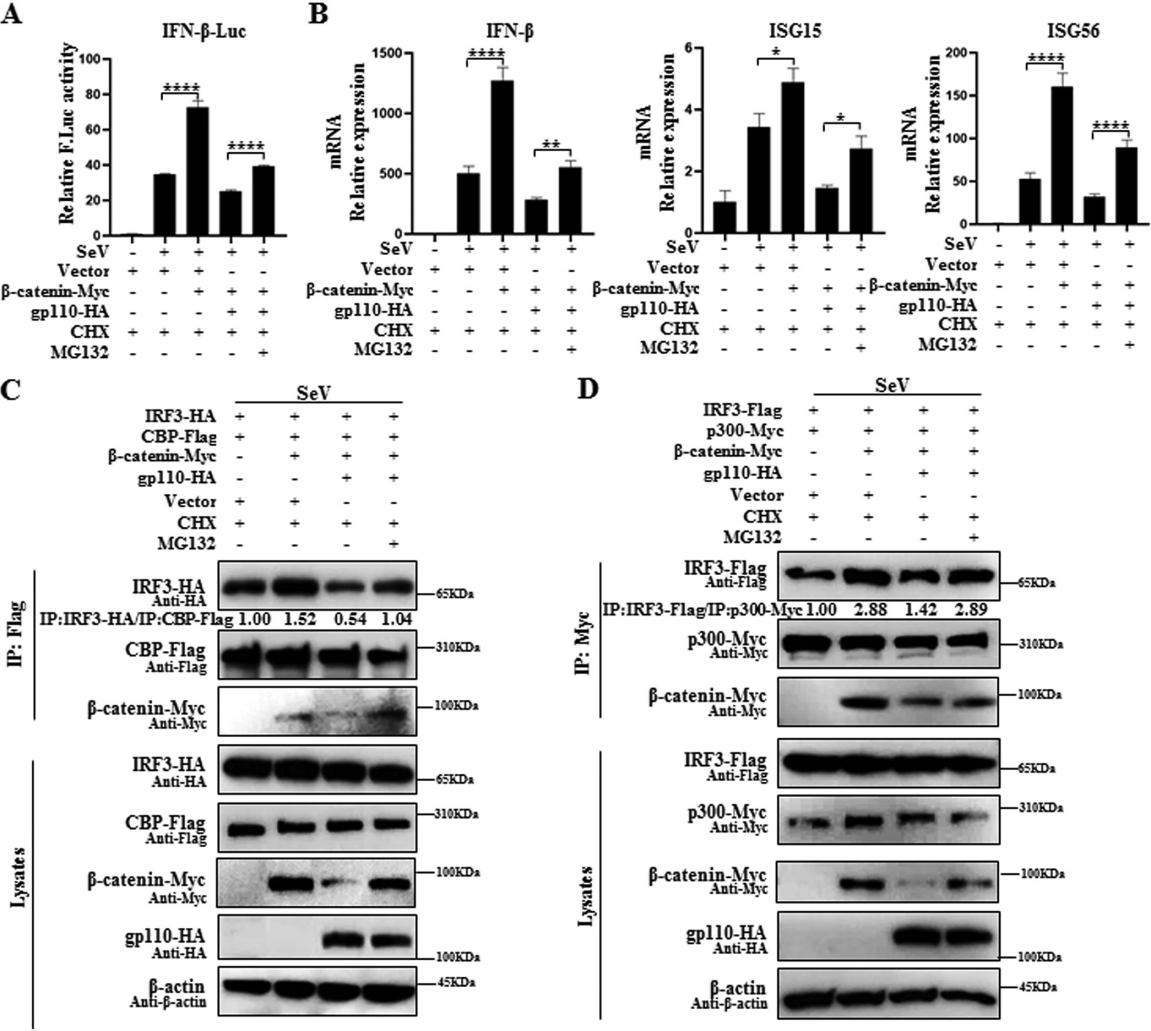
β-Catenin-mediated IFN-β production is antagonized by gp110. (A) One hundred nanograms of IFN-β-Luc and 10 ng of the internal reference pRL-TK were cotransfected with gp110-HA expression plasmid or vector into HEK293T cells along with or without β-catenin-Myc expression plasmid. At 24 h posttransfection, cells were infected with or without 100 HAU/mL SeV for 16 h. Cells were treated with CHX (50 μg/mL) alone or together with or without MG132 (20 μM) for 4 h. Cell lysates were collected, and luciferase activity was detected by DLR. (B) HEK293T cells were cotransfected with the expression plasmids of β-catenin-Myc and gp110-HA or vector. At 24 h posttransfection, cells were infected with or without 100 HAU/mL SeV for 16 h. Cells were treated with CHX (50 μg/mL) alone or together with or without MG132 (20 μM) for 4 h. Cells were collected, and total RNA was extracted. Real-time qPCR was used to detect the mRNA expression of *IFN-β*, *ISG15*, *ISG56*, and *GAPDH*. Data are presented as mean ± SD, and statistical analysis was performed using a Student’s *t* test; *, 0.01 < *P* < 0.05; **, 0.001 < *P* < 0.01; ****, *P* < 0.0001. (C and D) gp110-HA expression plasmid or vector was cotransfected with the expression plasmid combination of IRF3-HA/CBP-Flag (C) or IRF3-Flag/p300-Myc (D) into HEK293T cells along with or without β-catenin-Myc expression plasmid. At 24 h posttransfection, cells were infected with or without 100 HAU/mL SeV for 16 h. Cells were treated with CHX (50 μg/mL) alone or together with or without MG132 (20 μM) for 4 h, collected, and lysed. The samples were then used for Co-IP analysis with mouse anti-Flag MAb (C) or mouse anti-Myc MAb (D). Immunoprecipitated proteins were then resolved by 10% SDS-PAGE, and WB was performed with mouse anti-Flag MAb, mouse anti-Myc MAb, and mouse anti-HA MAb. β-Actin was used as the loading control. The gray analysis was calculated using ImageJ.

To explore whether the degradation of β-catenin affects activation of the IFN-β signaling pathway, HEK293T cells were cotransfected with IFN-β-Luc and gp110 expression plasmid or vector along with β-catenin expression plasmid, and cells were treated with SeV and cycloheximide (CHX) in the presence or absence of the proteasome inhibitor MG132. DLR assays showed that β-catenin could enhance SeV-induced IFN-β promoter activation, illustrating that IFN-β production requires β-catenin, while gp110 inhibited this activation, and this inhibition could be restored by MG132 (Fig. 7A). In addition, qPCR also demonstrated that β-catenin could increase the mRNA expression of *IFN-β* and its downstream antiviral genes (Fig. 7B), indicating that gp110 can inhibit β-catenin-mediated activation of IFN-β.

To evaluate whether gp110-mediated β-catenin degradation can affect its interaction with IRF3 or CBP/p300, HEK293T cells were cotransfected with β-catenin and the expression plasmid combination of IRF3-Flag/p300-Myc or IRF3-HA/CBP-Flag along with gp110 expression plasmid or vector. Cells were then treated with SeV and CHX in the presence or absence of the proteasome inhibitor MG132. Co-IP showed that gp110 could inhibit β-catenin-dependent increased interactions between IRF3 and CBP (Fig. 7C) or between IRF3 and p300 (Fig. 7D) due to the expression of β-catenin being reduced, but this inhibition was disrupted in the presence of MG132. Collectively, these results suggest that gp110 promotes the degradation of β-catenin to weaken the link between CBP/p300 and IRF3, thereby reducing IFN-β production.

### gp110 disrupts IKKi-mediated activation of NF-κB.

**FIG 8:**
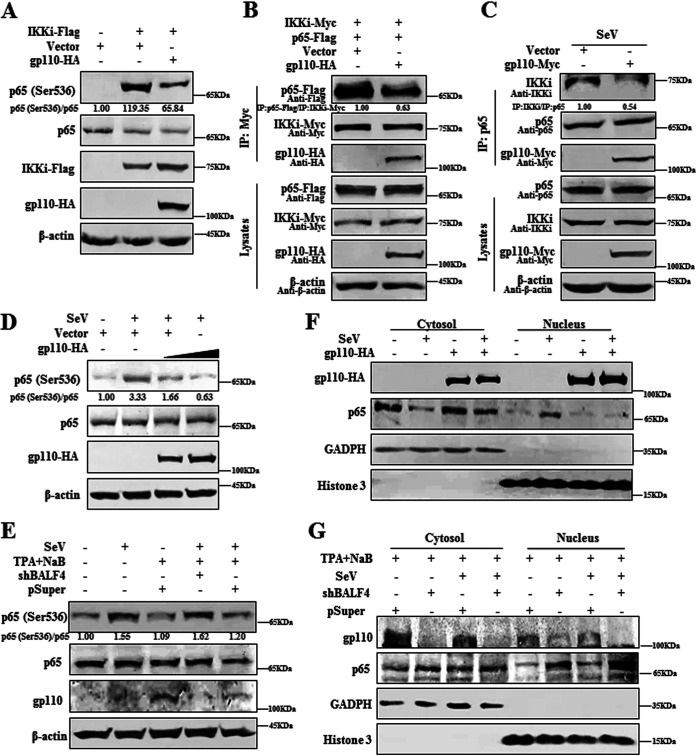
gp110 disrupts IKKi-mediated activation of the NF-κB pathway. (A) HEK293T cells were transfected with gp110-HA expression plasmid or vector along with or without IKKi-Flag expression plasmid to activate the phosphorylation of p65 (Ser536). At 24 h posttransfection, the cell lysates were collected for WB with rabbit anti-p65 pAb, mouse anti-Flag MAb, and mouse anti-HA MAb. Rabbit anti-p65 (Ser536) pAb was used to detect the phosphorylation of p65, and β-actin was used as the loading control. (B and C) gp110-HA expression plasmid or vector was individually transfected into HEK293T cells (C) or cotransfected with the plasmid combination of IKKi-Myc/p65-Flag into HEK293T cells (B). At 24 h posttransfection, cells were infected with or without 100 HAU/mL SeV for 16 h, collected, and lysed. The samples were then used for Co-IP analysis with mouse anti-Myc MAb (B) or rabbit anti-p65 pAb (C). Immunoprecipitated proteins were then resolved by 10% SDS-PAGE, and WB was performed with mouse anti-Flag MAb, mouse anti-Myc MAb, and mouse anti-HA MAb. Rabbit anti-IKKi pAb and rabbit anti-p65 pAbs were used to detect the expression of endogenous IKKi and p65 (C), respectively, and β-actin was used as the loading control. (D) HEK293T cells were transfected with different concentrations of gp110-HA expression plasmid or vector. At 24 h posttransfection, cells were infected with or without 100 HAU/mL SeV for 16 h. Cell lysates were collected for WB with rabbit anti-p65 pAb and mouse anti-HA MAb. Rabbit anti-p65 (Ser536) pAb was used to detect the phosphorylation of p65, and β-actin was used as the loading control. (E) Hone1-EBV cells were transfected with the expression plasmid of shBALF4 or pSuper vector. At 24 h posttransfection, cells were treated with or without TPA (40 ng/mL) and NaB (3 mM) for 24 h to induce lytic EBV infection. Cells were then infected with or without 100 HAU/mL SeV for 16 h, and cell lysates were collected for WB with mouse anti-gp110 MAb, rabbit anti-p65 pAb, and rabbit anti-p65 (Ser536) pAb. β-Actin was used as the loading control. (F) HEK293T cells were transfected with gp110-HA expression plasmid or vector. At 24 h posttransfection, cells were infected with or without 100 HAU/mL SeV for 16 h. Cell lysates were harvested for cellular fractionation, and WB was performed with mouse anti-HA MAb and rabbit anti-p65 pAb. (G) Hone1-EBV cells were transfected with the expression plasmid of shBALF4 or pSuper vector. At 24 h posttransfection, cells were treated with or without TPA (40 ng/mL) and NaB (3 mM) for 24 h to induce lytic EBV infection. Cells were infected with or without 100 HAU/mL SeV for 16 h, and cell lysates were collected for WB with mouse anti-gp110 MAb and rabbit anti-p65 pAb. Here, GAPDH was used as the cytosol marker, and histone 3 was used as the nucleus marker. The gray analysis was calculated using ImageJ.

In the above Co-IP analysis, it is found that gp110 interacts with IKKi and inhibits its kinase activity, but it has no effect on the activation of IRF3 in the signaling pathway (Fig. 3 to 5). In addition to regulating IRF3 activity, IKKi is also critical for NF-κB activation, which mediates p65 (Ser536) phosphorylation in response to proinflammatory signals and viral infection (6, 7). To assess whether gp110 can interfere with IKKi regulation of the NF-κB pathway, gp110 expression plasmid or vector was transfected into HEK293T cells along with IKKi expression plasmid to activate the phosphorylation (Ser536) of p65, and WB showed that gp110 could inhibit IKKi-induced p65 phosphorylation (Fig. 8A). To identify its specific mechanism, vector or gp110 expression plasmid was individually transfected or cotransfected with the plasmid combination of IKKi-Myc/p65-Flag into HEK293T cells. Cells were then treated with SeV, and Co-IP showed that gp110 could attenuate the overexpressed (Fig. 8B) and endogenous (Fig. 8C) interactions between IKKi and p65.

To further prove this result, HEK293T cells were transfected with vector or gp110 expression plasmid and treated with SeV, and WB again confirmed that gp110 could dose-dependently decrease the phosphorylation of p65 when the RLR signaling pathway was stimulated by virus (Fig. 8D). When the Hone1-EBV cells were transfected with pSuper or shBALF4 expression plasmid and treated with TPA/NaB and SeV, shBALF4 could increase SeV-induced p65 phosphorylation during EBV lytic infection compared with the pSuper control plasmid (Fig. 8E).

To estimate whether gp110 can block SeV-triggered nuclear translocation of p65, HEK293T cells were transfected with vector or gp110 expression plasmid and treated with SeV, then cellular fractionation was performed. As a result, SeV induced the nuclear accumulation of p65, but this accumulation was inhibited in the presence of gp110 (Fig. 8F). To finally verify this result, Hone1-EBV cells were transfected with pSuper vector or shBALF4 expression plasmid and treated with TPA/NaB and SeV. Compared with the pSuper control, shBALF4 could increase SeV-induced nuclear translocation of p65 during lytic EBV infection (Fig. 8G). Taken together, these data reveal that the attenuated K63-linked polyubiquitination of IKKi by gp110 results in restricting IKKi activity, which impairs IKKi activation of the RLR pathway mediating NF-κB function and weakening IFN-I-mediated antiviral immunity.

## DISCUSSION

EBV is a large DNA virus that encodes over 80 proteins for complex interactions with the host. The EBV *BALF4* gene encodes gp110, also called glycoproteins B (gB), a glycoprotein composed of 857 amino acids (aa) and three domains, including a large N-terminal ectodomain structure (685 aa) containing 9 potential N-linked glycosylation sites, 3 hydrophobic regions, and a region of 104 aa located at the C-terminal tail (74, 75). gB is conserved in herpesviruses, and some homologous proteins of gp110 have been reported, such as HSV-1 UL27 (gB) (29, 30), murine herpesvirus 68 (MHV-68) gB (76), human cytomegalovirus (HCMV) UL55 (77), and Kaposi sarcoma-associated herpesvirus (KSHV) open reading frame 8 (ORF8) (78), which are crucial for the viral replication cycle (79–82). Additionally, the interaction of gB with heparin sulfate proteoglycan is involved in initial adhesion to the cell surface (83–85).

HSV-1 gB interacts with Toll-like receptor 2 (TLR2) to trigger the MyD88/TRAF6-dependent signaling pathway, leading to the activation of NF-κB and the induction of inflammatory cytokine production (86). When the host is invaded by a virus, IFN-I production is an important mechanism to establish a rapid and effective innate immunity. Numerous studies have shown that IFN is fundamental in limiting EBV replication and infection (87, 88). However, it is not clear whether gp110 can negatively regulate IFN-I production. In this study, we reported that gp110 overexpression could inhibit IFN-β promoter activity as well as the transcription of downstream antiviral genes to promote viral proliferation. When gp110 was knocked down by specific short hairpin RNA (shRNA), gp110-inhibited IFN-β production was restored.

Here, superinfection with SeV was performed in nasopharyngeal carcinoma epithelial Hone1 cells latently infected with EBV (Hone1-EBV) (89). Transcription of *IFN-β*, *ISG15*, and *ISG56* was relatively low when EBV was induced into lytic infection after treatment of Hone1-EBV cells with TPA and NaB, likely due to the fact that detection of *IFN-β* transcription was performed after EBV lytic infection for 24 h. This was designed to ensure that gp110 could reach a high expression level, as the peak expression of gp110 is around 24 h after the initiation of EBV lytic infection (90). However, EBV also encodes several late proteins by this time that aid in immune evasion, such as some reported proteins (BGLF4 [91], BPLF1 [92], BGLF2 [93], BFRF1 [20], and so on) and other unknown EBV products. Thus, *IFN-β* showed a relatively low transcript level in the presence of these EBV products. In addition, Gujer and colleagues showed the antiviral genes also showed a low transcriptional level after EBV lytic infection in peripheral blood mononuclear cells for 24 h (94). Our previous study also confirmed that the transcription of IFN-I and ISGs was very low during EBV lytic infection for about 24 h in Hone1-EBV cells (20).

To better demonstrate the role of gp110 in inhibiting the RLR signaling pathway, the expression of gp110 was knocked down after inducing EBV lytic infection in Hone1-EBV cells. SeV was then added to the cells to amplify the activation effect of the signaling pathway to make the change trend more evident, which can reflect the function of gp110 in suppressing the RLR signaling pathway during lytic infection; this experimental method is commonly used (20). In fact, a similar method was also applied in our previous study and was used to analyze the inhibitory effect of the tegument protein BGLF2 on the NF-κB signaling pathway and its downstream inflammatory factors during EBV lytic infection of Hone1-EBV cells, followed by knockdown of the expression of BGLF2 and stimulation of cells with TNF-α (24).

It is known that transcriptional activation of IFN-β is associated with different transcription factors, such as IRF3 or NF-κB (56), binding to diverse regulatory regions in the IFN-β promoter. We found that the expression of gp110 represses SeV-mediated promoter activity of both IRF3 and NF-κB branches, and Co-IP experiments showed that gp110 interacts with IKKi. Ubiquitination is a common posttranslational modification, and several regulatory molecules in the IFN-β signaling pathway require ubiquitination (45, 46). Studies have shown that K63-linked polyubiquitination of IKKi can promote the activation of IRF3 (42, 47). We found that gp110 could interact with IKKi and suppresses its K63-linked polyubiquitination but had no effect on its K48-linked polyubiquitination; thereby, gp110 inhibits IKKi-activated IRF3 phosphorylation. Many viral proteins have been reported to interfere with IRF3 activation (49, 95, 96). Surprisingly, gp110 did not affect the phosphorylation, dimerization, or nuclear transport of IRF3 under the stimulation of SeV, which may be due to the redundant roles of TBK1 and IKKi in the IFN-I production pathway (67, 97), and gp110 only acts on IKKi.

Following translocation of IRF3 into the nucleus, CBP and/or p300 is recruited, and this complex then binds to PRDIII-I in the IFN-β promoter to advance IFN-β production (70). It is well known that β-catenin is a key effector of Wnt signaling and plays a vital role in cell growth and differentiation as well as virus-induced IFN-β transcription (69). β-Catenin can interact with IRF3 to promote the recruitment of p300 to the IFN-β promoter. Thus, β-catenin-deficient cells produce less IFN-β after infection, and the antiviral innate immune response is subsequently attenuated (72). In addition, viral infection can stimulate the induction of the β-catenin-IRF3-CBP/p300 complex, which also contributes to IFN-β production (13, 14). In this study, gp110 interacted with β-catenin and promoted its K48-linked polyubiquitination, but addition of the proteasome inhibitor MG132 restored its expression. Therefore, gp110 promoted β-catenin K48-linked polyubiquitination degradation via the proteasome, leading to a decrease in the role of β-catenin in promoting IFN-β production and the expression of downstream antiviral genes. The interaction between CBP/p300 and IRF3 was also attenuated, and the reduced formation of the β-catenin-IRF3-CBP/p300 complex resulted in decreased IFN-β production. To date, no study has shown that gp110 possesses ubiquitinase activity, and it does not contain a corresponding domain itself. Thus, gp110 may be associated with some ubiquitin ligases to accomplish ubiquitination, but this needs to be investigated in a future study. For example, EBV BGLF2 can interact with the E3 ubiquitin ligase cullin 1 to promote its recruitment of STAT2, which eventually causes its degradation (98).

NF-κB and IRF are two families of transcription factors closely related to viral infection-induced downstream gene transcription (99). In the above results, we found that gp110 interacted with IKKi and disturbed the effect of IKKi on activating IRF3. However, the function of IRF3 was not decreased when the pathway was induced by SeV. Several studies have shown that in addition to regulating the activation of IRF3, IKKi is also involved in regulating the activation of NF-κB (6). We found that IKKi-mediated activation of p65 was inhibited by gp110, suggesting that gp110 negatively regulates the NF-κB pathway through IKKi. Moreover, gp110 weakened the interaction between IKKi and p65, which also inhibited SeV-induced phosphorylation and nuclear translocation of p65. Simultaneously, SeV-induced phosphorylation and nuclear translocation of p65 increased when gp110 was knocked down during EBV lytic infection. A large amount of gp110 is localized in the endoplasmic reticulum and nuclear membrane when it is expressed in cells (26, 100), and we could also see by IFA that a part of gp110 was localized to the nuclear membrane (Fig. 5C). In Fig. 8F, a cellular fractionation protein extraction kit was used to sufficiently swell the cells under low-osmolarity conditions. The cell membrane was then disrupted, and the cytoplasmic proteins were released. The nuclear pellet was obtained by centrifugation, and nuclear proteins were extracted using high-salt nuclear protein extraction reagent. Since a part of gp110 was localized in the nuclear membrane, this part of gp110 was separated in the nuclear fraction during the cellular fractionation process; thus, a certain amount of gp110 was also present in the nucleus, as detected by WB. The cellular fractionation results of the cytoplasmic marker glyceraldehyde phosphate dehydrogenase (GAPDH) and nuclear marker histone 3 also showed that there was no problem with the experimental technique. Therefore, the experimental results were correct (Fig. 5C and 8F and G). Consequently, the K63-linked polyubiquitination of IKKi is attenuated by gp110, which results in lessened IKKi activity and impairs IKKi activation of the NF-κB pathway, thereby diminishing the production of inflammatory cytokines and decreasing antiviral immunity.

**FIG 9:**
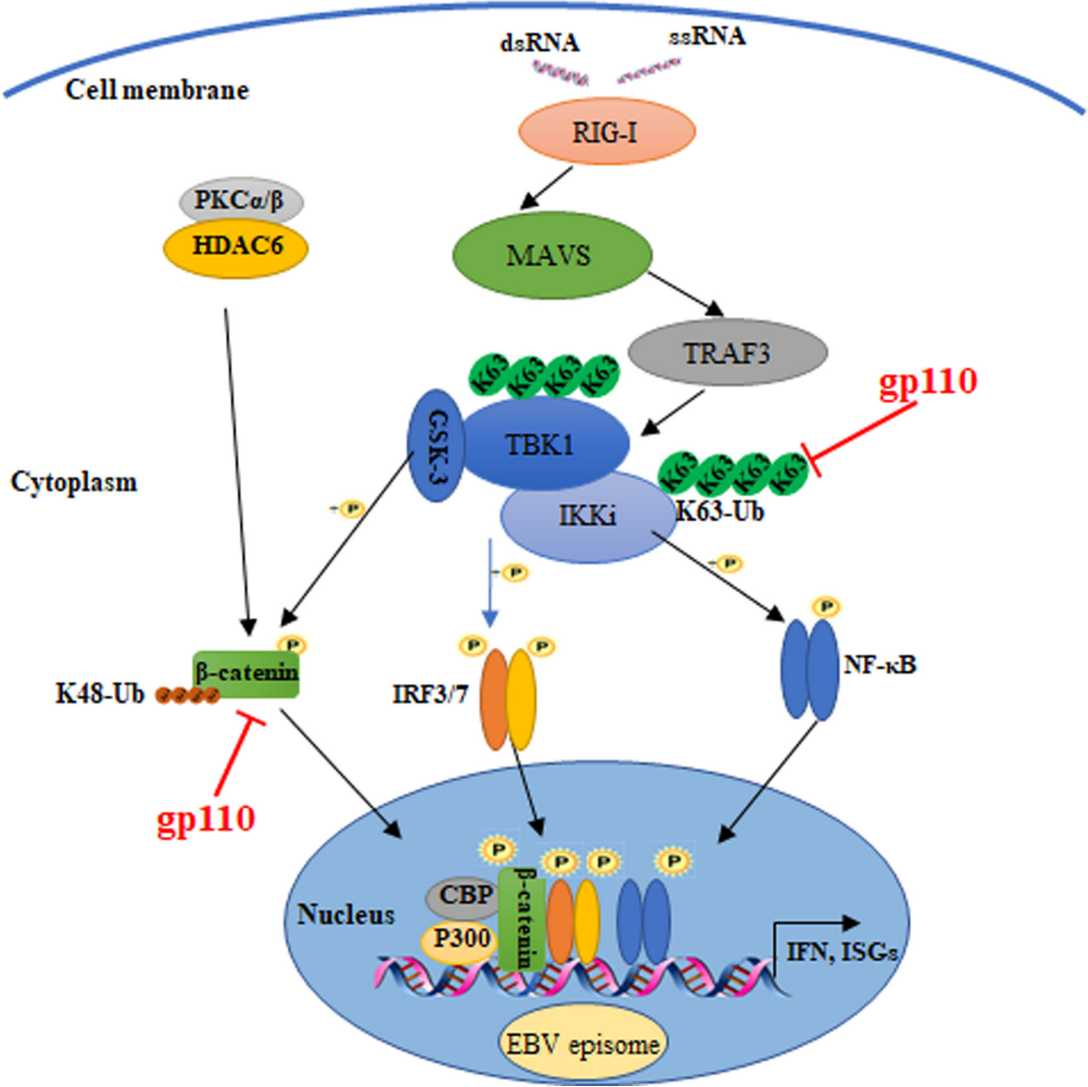
Schematic diagram of the molecular mechanism by which gp110 inhibits the RLR signaling pathway. In the RLR signaling pathway, RIG-I and MDA5 recognize dsRNA or 5′-ppp ssRNA to induce polymerization of the mitochondrial adaptor protein MAVS. MAVS then recruits TRAFs for the activation of the kinases TBK1 and IKKi. Subsequently, the transcription factors IRF3 and IRF7 are phosphorylated by kinases then dimerize and transport to the nucleus. At the same time, β-catenin is deacetylated by histone deacetylase 6 (HDAC6) and phosphorylated by glycogen synthase kinase 3 (GSK-3) after cells are stimulated. β-Catenin then forms a complex with IRF3 and CBP/p300 to induce the transcription of IFN-β. When EBV invades the cell, its genome can exist in the nucleus as an episome. Here, gp110 was found to interact with IKKi and inhibit its K63-linked polyubiquitination to attenuate IKKi-mediated NF-κB activation. Furthermore, gp110 binds to β-catenin to promote its proteasomal degradation through K48-linked polyubiquitination and reduce the formation of the β-catenin-IRF3-CBP/p300 conformer, resulting in decreased IFN-β production.

Taken together, we found that gp110 is a negative regulator of host antiviral innate immunity. gp110 interacts with IKKi to attenuate IKKi-mediated activation of NF-κB, thereby inhibiting IKKi-induced p65 phosphorylation and nuclear transport. Furthermore, the interaction between gp110 and β-catenin promotes its K48-linked polyubiquitination-dependent degradation via the proteasome, which constrains the formation of the β-catenin-IRF3-CBP/p300 conformer, thus reducing IFN-β production (Fig. 9). These data expand our knowledge of the mechanisms by which EBV interacts with the host and facilitate our better understanding of the virus as well as rational vaccine design and antiviral drug development against EBV infection.

## MATERIALS AND METHODS

### Cells culture, viral infection, antibodies, and reagents.

Human embryonic kidney 293T (HEK293T) cells and HeLa cells were cultured in Dulbecco’s modified minimum essential medium (DMEM; Gibco-BRL) supplemented with 10% fetal bovine serum (FBS; Gibco-BRL) and 100 U/mL penicillin and streptomycin. Hone1-EBV cells (a gift from Sai Wah Tsao, University of Hong Kong) are an EBV^+^ nasopharyngeal carcinoma cell line cultured the same as HEK293T cells, except that RPMI 1640 (Thermo Fisher Scientific) was used. Sendai virus (SeV), an activator of the RLR pathway, was amplified in chicken embryos and preserved at −80°C in our laboratory. Green fluorescent protein (GFP)-tagged vesicular stomatitis virus (VSV) (VSV-GFP) was maintained in our laboratory, GFP-tagged wild-type (WT) herpes simplex virus 1 (HSV-1) F strain (HSV-1-GFP) was offered by Chunfu Zheng (Department of Microbiology, Immunology and Infectious Diseases, University of Calgary) and was reproduced in Vero cells and preserved in our laboratory (101). Mouse anti-Flag, anti-Myc, and anti-hemagglutinin (HA) monoclonal antibodies (MAbs) and rabbit anti-β-catenin polyclonal antibody (pAb) were purchased from Abmart, rabbit anti-Flag and anti-HA MAbs were provided by Affinity Biosciences, and rabbit anti-Myc MAb was offered by Bioss. Rabbit anti-ubiquitin MAb, anti-IRF3 (Ser396), and anti-p65 (Ser536) phosphorylated pAbs, alkaline phosphatase (AP)-labeled goat anti-mouse IgG, and goat anti-rabbit IgG were purchased from Cell Signaling Technology. Mouse anti-CBP and anti-p300 pAbs were supplied from Santa Cruz, and mouse anti-gp110 MAb was offered by GeneTex. Rabbit anti-IRF3, anti-p65, and anti-histone-H3 pAbs, mouse anti-GAPDH MAb, and IgG negative-control antibody were obtained from Proteintech, rabbit anti-IKKi pAb was acquired from Abcam, Cy5-conjugated goat anti-rabbit IgG and fluorescein isothiocyanate (FITC)-conjugated goat anti-mouse IgG were purchased from BBI Life Sciences, and rabbit anti-β-actin MAb was purchased from Abclonal Technology. Additionally, 12-*O*-tetradecanoylphorbol-13-acetate (TPA) and sodium butyrate (NaB) were purchased from Biotechnology and Beyotime, respectively, to induce EBV lytic infection in Hone1-EBV cells. Cycloheximide (CHX) and MG132 were acquired from Sigma and InvivoGen, respectively.

### Plasmid construction.

All enzymes used for cloning were purchased from Thermo Fisher Scientific, except for T4 DNA ligase (Vazyme). To construct the Myc-tagged gp110 expression plasmid, the open reading frame (ORF) of gp110 encoding the *BALF4* gene was amplified by PCR using bacterial artificial chromosome (BAC) DNA of the EBV Akata strain as the template, with the forward primer 5′-TTAAGCTTCCGAATTCATGACTCGGCGTAGGGTGCTAAG-3′ and the reverse primer 5′-TTGCGGCCGCAGGATCCAAAAACTCAGTCTCTGCCTCCCC-3′. The product was then purified, digested with EcoRI and BamHI, and ligated into the pMyc-N1 vector (regenerated from pEYFP-N1, Clontech) to obtain the expression plasmid gp110-Myc. Furthermore, expression plasmids of BGLF4-HA, gp110-HA, and shBALF4 were constructed in our previous study (24, 39). β-Catenin-Myc, RIG-I-Flag, IKKi-Myc, TBK1-Myc, IRF7-HA, CBP-Flag, p300-Myc, and WT ubiquitin expression plasmid (Ub-WT-HA) were donated by Chunfu Zheng (102), and Ub-K48-HA and Ub-K63-HA expression plasmids were gifts from Hongbing Shu (103). Plasmids expressing ISRE-Luc (104), (PRDIII-I)4-Luc (105), IFN-β-Luc (106), NF-κB-Luc, RL-TK (107), IKKi-Flag (104), IRF3-HA (108), IRF3-Flag (109), p65-Flag (110), MAVS-Flag, IKKi-Myc, TRAF3-Flag, and TBK1-Flag (111) were described in our previous study (24, 112).

### Transfection and dual-luciferase reporter (DLR) assays.

Transfection and DLR assays were performed as previously described (66, 86). HEK293T cells or Hone1-EBV cells plated in 24-well dishes (Corning) with a confluency of about 80% were cotransfected with 100 ng of reporter plasmid (IFN-β-Luc, [PRDIII]4-Luc, ISRE-Luc, or NF-κB-Luc) and 10 ng of pRL-TK (*Renilla* luciferase reporter plasmid) to standardize transfection efficiency, along with the indicated amounts of expression plasmid(s). At 24 h posttransfection, TPA (40 ng/mL) and NaB (3 mM) were added to the cells for 24 h to induce EBV lytic infection. Cells were then infected with or without 100 hemagglutinin units (HAU)/mL SeV for 16 h to activate the RLR pathway. Cell lysates were collected, and the reporter plasmid luciferase activity was detected using a specific DLR kit (Promega, Madison, WI, USA). Protein expression of the transfected plasmid(s) was detected by Western blotting (WB). The results (firefly luciferase activity divided by *Renilla* luciferase activity) were expressed as the mean and standard deviation (SD) from three replicate experiments.

### Coimmunoprecipitation (Co-IP) assays.

Co-IP assays were performed as previously described (113, 114). Hone1-EBV cells were treated with TPA (40 ng/mL) and NaB (3 mM) for 24 h to induce EBV lytic infection, or HEK293T cells were transfected with the indicated plasmid(s) for 24 h. After EBV infection or plasmid transfection, cells were collected and lysed in RIPA lysis buffer (Beyotime) on ice for 30 min. The supernatant was subsequently incubated with 3 μg of the indicated Ab(s) or nonspecific IgG at 4°C overnight. A 1:1 slurry of protein A/G plus agarose (Beyotime) was added for 4 h. The bead-antibody-protein complexes were then subjected to sodium dodecyl sulfate-polyacrylamide gel electrophoresis (SDS-PAGE) and WB.

### WB.

WB was performed as described previously (115–117). In brief, the protein samples were subjected to 10% SDS-PAGE and transferred to nitrocellulose membranes (Pall, Port Washington, NY, USA). Membranes were then incubated with the indicated primary Abs, followed by AP-labeled goat anti-rabbit IgG or goat anti-mouse IgG secondary Ab. Finally, the Ab-specific binding bands were colored by 5-bromo-4-chloro-3-indolephosphoric acid/nitroblue tetrazole (BCIP/NBT; Biosharp, Shanghai, China), and the reaction was terminated by adding distilled water.

### Native PAGE.

Native PAGE was executed according to previous studies (20, 66). HEK293T cells plated in 6-well plates (Corning) were transfected with the indicated expression plasmid(s). At 24 h posttransfection, cells were infected with or without 100 HAU/mL SeV for 16 h and lysed with weak RIPA lysis buffer (Beyotime). Subsequently, the samples were subjected to nondenatured gel electrophoresis, and the protein was transferred to nitrocellulose membranes. WB analysis was performed, as described above.

### RNA isolation and real-time quantitative PCR (qPCR).

RNA isolation and real-time qPCR were accomplished as described previously (24, 118). In short, HEK293T cells or Hone1-EBV cells plated in 6-well plates were transfected with the indicated expression plasmid(s), and at 24 h posttransfection, cells were treated with or without TPA (40 ng/mL) and NaB (3 mM) for 24 h to induce EBV lytic infection. Cells were then infected with or without 100 HAU/mL SeV for another 16 h and collected, and total RNA was extracted by using a TRIzol kit (GenStar). Reverse transcription was performed using a Star Sriptll first-strand cDNA kit (GenStar). The reverse-transcribed cDNA was used as the real-time qPCR template, and target gene expression was detected with specific primers using the SYBR green procedure and a CFX96 real-time PCR detection system (Bio-Rad, Hercules, CA, USA). The expression levels of target genes were normalized with that of the housekeeping gene *GAPDH*, and the relative fold value is the ratio of the expression value in each reaction mixture to the value for vector-transfected cells. The following primer sequences were used in this study: 5′-GCTTCGTGACCAACACAACC-3′ (forward) and 5′-GTAATGGCTTCCTGGCCCTT-3′ (reverse) for gp110, 5′-ATGACCAACAAGTGTCTCCTCC-3′(forward) and 5′-GGAATCCAAGCAAGTTGTAGCTC-3′ (reverse) for *IFN-β*, 5′-TGGACAAATGCGACGAACCTC-3′ (forward) and 5′-TCAGCCGTACCTCGTAGGTG-3′ (reverse) for *ISG15*, 5′-TACAGCAACCATGAGTACAA-3′(forward) and 5′-TCAGGTGTTTCACATAGGC-3′ (reverse) for *ISG56*, 5′-GAAAGCAGCAAAGAGGCACT-3′ (forward) and 5′-TTTCACCAGGCAAGTCTCCT-3′ (reverse) for *IL-6*, 5′-GGTGCAGTTTTGCCAAGGAG-3′ (forward) and 5′-TTCCTTGGGGTCCAGACAGA-3′ (reverse) for *IL-8*, and 5′-AGGTCGGAGTCAACGGATTTG-3′ (forward) and 5′-TGTAAACCATGTAGTTGAGGTCA-3′ (reverse) for *GAPDH* (20). All the real-time qPCR assays were repeated three times, and the results show the mean of representative experiments.

### Cellular fractionation.

HEK293T cells or Hone1-EBV cells plated in 60-mm cell culture dishes were transfected with the indicated expression plasmid(s) or vector, and at 24 h posttransfection, cells were treated with or without TPA (40 ng/mL) and NaB (3 mM) for 24 h to induce lytic EBV infection. Cells were then infected with or without 100 HAU/mL SeV for another 16 h to activate host innate immunity. Cells were then collected, and the cytoplasm was separated from the nucleus using a cellular fractionation kit (Beyotime). Finally, the isolated cell components were subjected to SDS-PAGE and WB with the indicated Abs.

### Indirect immunofluorescence assay (IFA).

Indirect IFAs were performed as previously described (119). HeLa cells were transfected with vector or gp110-HA expression plasmid, and at 24 h posttransfection, cells were infected with or without 100 HAU/mL SeV for 16 h. Cells were then fixed in 4% paraformaldehyde (Beyotime Biotechnology) for 30 min and permeabilized with 0.1% Triton X-100 (Notlas) for 10 min. Subsequently, cells were incubated with the primary Abs mouse anti-HA MAb and rabbit anti-IRF3 pAb overnight, followed by incubation with secondary Abs FITC-labeled goat anti-mouse IgG (green) and Cy5-labeled goat anti-rabbit IgG (red) for 1 h at room temperature. Cells were then counterstained with Hoechst 33342 (Millipore Sigma) to show the nucleus. The samples were analyzed by confocal microscopy (SP8, Leica Microsystems, Buffalo Grove, IL, USA).

### Statistical analysis.

The experiment results involved in the statistical analysis in our article were compared between two groups. Data are presented as mean ± SD. A Student’s *t* test was applied for comparisons between two groups in GraphPad Prism 8 software, which is commonly used in the literatures (120–125). Differences were considered significant when the *P* value was less than 0.05 and are marked with asterisks in the figures; *, 0.01 < *P* < 0.05; **, 0.001 < *P* < 0.01; ***, 0.0001 < *P* < 0.001; ****, *P* < 0.0001.
